# Point-of-care diagnostics: extending the laboratory network to reach the last mile

**DOI:** 10.1097/COH.0000000000000351

**Published:** 2017-08-09

**Authors:** Paul K. Drain, Christine Rousseau

**Affiliations:** a Department of Global Health; b Department of Medicine; c Department of Epidemiology, University of Washington, Seattle, Washington; d Department of Surgery, Massachusetts General Hospital, Harvard Medical School, Boston, Massachusetts; e Bill and Melinda Gates Foundation, Seattle, Washington, USA

**Keywords:** CD4^+^ cell count, centralized laboratory, HIV self-testing, HIV viral load, HIV/AIDS, point-of-care test, resource-limited settings

## Abstract

**Purpose of review:**

More point-of-care (POC) diagnostic tests are becoming available for HIV diagnosis and treatment in resource-limited settings. These novel technologies have the potential to foster decentralized HIV care and treatment for the benefit of clinical laboratories, HIV clinics, and HIV-infected patients. There continue to be many business, technological, and operational challenges that limit product development and regulatory approval, which limits products available for the required operational and cost-effectiveness studies and delays policy adoption and implementation.

**Recent findings:**

Although the rapid HIV diagnostic test has been widely successful, the pathways for POC CD4^+^ cell count and HIV viral load assay analyzers have been more challenging. We describe significant hurdles for product development, approval, and implementation, which include the business case, technical development, clinical impact, and integrating laboratory and clinical networks.

**Summary:**

The objective of this review is to highlight the obstacles for developing and implementing appropriate strategies for POC HIV testing assays to improve the clinical services for HIV-infected patients in resource-limited settings.

## INTRODUCTION

Approximately 36 million people are currently living with HIV, and nearly half of them are receiving chronic antiretroviral therapy (ART) [1]. The Joint United Nations Programme on HIV/AIDS (UNAIDS) has committed to achieving the 90-90-90 treatment targets by 2020, whereby 90% of people living with HIV know their HIV status, 90% of people with diagnosed HIV infection receive sustained ART, and 90% of people receiving ART have viral load suppression [2]. Given that South Africa, the country with the most HIV-infected people worldwide, had only achieved only 29% virologic suppression of HIV in a low-income township by 2013 [3], there remains much work to be done in diagnosing, treating, and monitoring HIV-infected individuals. Globally, innovative solutions will be required to maximize the use of limited public health infrastructure, human resource, and financing.

In 2013, the WHO-recommended routine HIV viral load testing as a more effective way of monitoring patients on ART [4], and several countries, including South Africa, have adopted viral load monitoring. However, viral load and early infant diagnostic tests utilize access to centralized reference laboratories, which require significant management and operational infrastructure. Furthermore, high HIV incidence throughout sub-Saharan Africa has increased pressure on HIV clinics to rapidly expand accessibility to ART. As clinics are already overburdened and understaffed, many locations are experiencing long patient waiting times and poor retention in care. The strains placed on HIV clinicians and laboratories have detracted resources from identifying new patients, initiating more people on ART, and appropriately switching patients with treatment failure to second-line ART regimens.

Among countries that have developed centralized laboratory capacity for HIV testing, the capacity has been poorly utilized [5] and access has remained low [6]. In a WHO survey of 127 member states, sufficient centralized laboratory capacity existed for at least four CD4^+^ cell count tests per HIV-infected person per year, but only 11% of the CD4^+^ testing capacity was utilized [5]. Similarly, laboratory capacity could support 0.44 HIV viral load tests per HIV-infected person per year, but only 36% of this capacity had been utilized. Compounding the problem of utilization, the Clinton Health Access Initiation (CHAI) found that only 50% of the laboratory-based HIV test results had been returned to the clinics in Mozambique, Malawi, and South Africa [7]. The underutilization of existing laboratory capacity, improper test reporting, along with misinterpretation and inaction at the clinical sites, suggest the persistence of major logistical and management challenges for centralized laboratories, reflecting a lost opportunity to provide better care.

Over 10 years ago, a similar scenario existed for diagnostic HIV ELISA-based antibody testing – a laboratory-based immunological assay was the only testing method available. The commercialization of a rapid HIV diagnostic test became a revolutionary point-of-care (POC) test, enabling decentralization of testing that ultimately increased the number of people living with HIV knowing their HIV status. Since 2007, when the WHO recommended routine HIV screening in healthcare settings, POC HIV testing has rapidly accelerated throughout the world. Between 2010 and 2014, over 600 million adults were screened with the rapid HIV test in 120 low-income and middle-income countries [8]. Unfortunately, in 2015, millions of HIV-infected people in need to ART still did not know their HIV status [1], suggesting more work needs to be done or innovative approaches to access.

Following the rapid HIV antibody test, other POC technologies have been developed to support decentralized care and treatment in the context of achieving the UNAIDS's 90-90-90 goals. The promise that POC diagnostics could be operated at the same location as a patient's clinical evaluation and return results during the visit have led to massive investments in technology development and deployment efforts to decentralize HIV testing. In this review, we describe the progress that has been made with POC HIV testing, the challenges that remain, and the future direction for reaching the last mile of HIV testing services. 

**Box 1:**
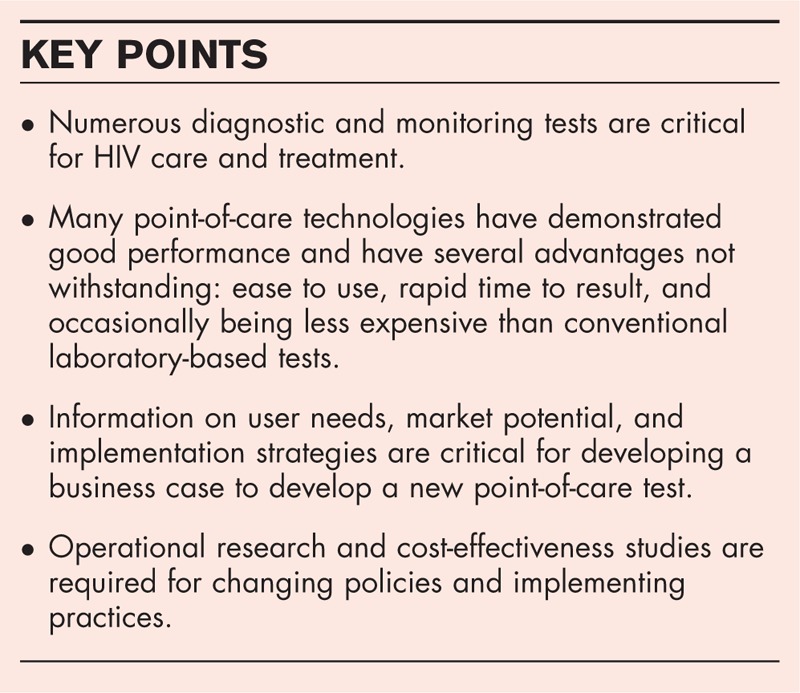
no caption available

## POINT-OF-CARE TECHNOLOGIES FOR HIV TESTING

Several new POC technologies for HIV testing have been designed to return test results at during the patient visit and at the site of clinical evaluation. Alere's PIMA machine (Alere Inc.; Waltham, Massachusetts, USA), launched in 2010, performs CD4^+^ cell count enumeration from a fingerprick of whole blood, and delivers a result within 20 min [9]. The fully integrated technology requires minimal operator steps, whereas the machine is small, lightweight, and portable. In 2014, Becton Dickinson released the FACSPresto (Becton Dickinson; Franklin Lakes, New Jersey, USA), a small, portable, low-cost POC CD4^+^ instrument that enables batch processing and a time-to-result in 4 min. New HIV viral load technologies are also available and many more are in the development pipeline [10,11]. These include benchtop devices that have a same-day turnaround time. Many are not fully integrated still requiring blood plasma separation prior to sample input. One example is Cepheid's Xpert HIV-1 viral load test, which measures HIV viral load with a dynamic range of 40–10 million copies/ml within 90 min on a GeneXpert instrument (Cepheid Inc.; Sunnyvale, California, USA) [12–14]. Overall, POC CD4^+^ instruments have achieved 25% of the market share in the developing world, paving the way for future technologies [15].

### Demonstrated accuracy and clinical benefit

As POC CD4^+^ technologies have been in use, several clinical accuracy and clinical effectiveness studies have been conducted to support their implementation. Alere's PIMA demonstrated good acceptability and feasibility in the field [16], and a recent meta-analysis reported excellent concordance between 11 803 paired venous and fingerprick blood specimens [17]. Similarly, Becton Dickinson's FACSPresto has demonstrated acceptable performance and test validation characteristics in multiple settings [18–20]. An implementation study has demonstrated that POC CD4^+^ testing can promote earlier ART initiation, reduce loss to follow-up rates, and improve linkage to HIV care and treatment [21].

Initial results of new HIV viral load tests have also been promising. The Alere viral load assay and the SAMBA semiquantitative test have proven accurate for viral load monitoring and early infant diagnosis in resource-limited settings [22–24]. Two studies have demonstrated that the Xpert HIV-1 viral load test was accurate among several HIV clades, when performed in a laboratory environment and compared with gold-standard viral load testing [25,26^▪^]. One clinic-based feasibility and validation study also demonstrated excellent accuracy for the Xpert HIV-1 viral load test performed in an HIV clinic in Durban, South Africa [27^▪^]. In this study, Xpert had strong correlation among a dynamic range of HIV viral load values (Spearman ρ = 0.94, *P* < 0.001), when compared with lab-based Roche Taqman v2.0 viral load testing (Roche Diagnostics, Risch-Rotkreuz, Switzerland). A Bland–Altman plot showed a mean difference between Taqman and Xpert results of −0.10 log copies/ml (95% limits of agreement −0.59 to 0.39), and all 12 observations from patients failing ART were detected by the Xpert assay.

As viral load tests that enable same-visit results have only recently become available, operational research studies are currently underway. However, predictive mathematical models have suggested that POC HIV viral load monitoring will enable more cost-effective delivery of ART [28^▪^]. The model results demonstrated that POC monitoring could provide significant savings by reducing follow-up clinic visits for stable HIV-infected patients receiving ART and redirecting clinic resources to HIV-infected patients with an unsuppressed viral load. Until a POC viral load technology is available that can accept whole blood specimens, dried blood spots can effectively be used for sample transport to a centralized laboratory facility to run the diagnostic test.

## REMAINING CHALLENGES FOR POINT-OF-CARE HIV DIAGNOSTICS

### Business case for product development

The path to reaching the commercial market with a new POC technology is a long and expensive process. Typically instrument product development may take 10 years and cost US$100 million from initial proof of concept to product launch (ref). The challenges along this continuum of development are numerous, and go far beyond the technical hurdles. In today's competitive market, companies are raising funds from smaller venture capital funds, while also pitching compelling business cases based on achieving sales goals within a shifting global market. Although few new POC technologies have been commercialized, many more companies have been unsuccessful in launching a promising new POC technology product for the global health market.

Product development is a long and costly process, and thus understanding the benefits of the future product or market is critical to supporting a continuation of a particular program. Technical challenges that arise during product development require frequent and iterative trade off decisions that may ultimately erode the original predicted benefit. One of the primary barriers to POC product development has been a lack of understanding of user needs in a way that can be used to inform product-development decision-making. An attempt to address this gap and inform the optimal characteristics of a cost-effective POC HIV viral load test and results from a mathematical modeling exercise indicated that the test failure and performance rates were the key characteristics to improve upon existing approaches to viral load testing [29]. Eventually, operational studies will be required to evaluate the impact of the POC HIV viral load monitoring intervention on overall coverage/access to testing, the likelihood of switching to a second-line ART regimen, and probability of implementing differentiated clinical care.

A primary challenge for product development of any new POC technology is forecasting the target market share. Using multiple sets of assumptions, CHAI estimates that POC HIV viral load testing will meet the needs for 7–36% of the global market share (Trevor Peter; personal communication). This wide range reflects the level of uncertainty surrounding product investment and adoption, which is due to limited data on the performance and clinical efficacy of the available and emerging products. Furthermore, the path to regulatory approval can be unclear and onerous. Although WHO has improved the transparency and efficiency of its prequalification process, the subsequent requirements for in-country registrations can be opaque and numerous. Efforts are underway to regionalize approvals and reduce the need for multicountry validations and registrations, but these regulatory improvements may take years to materialize. Finally, new policy is generally required before the adoption of a new POC device or technology, but the evidence required for those policy decisions are often unclear and the efforts to generate the required evidence may be long and uncoordinated. For these reasons, companies face difficulties in raising sufficient funds to either complete the product development or scale a promising new POC technology, and support from the global health community might be an essential element to the business case.

### Technical development

Sample collection has been an ongoing challenge for most POC technologies. Although Alere, Becton Dickinson, and Cepheid have each made significant advances in enabling effective sample collection in the field, sample collection issues remain. For example, Cepheid's Xpert HIV-1 viral load test has solved numerous technical barriers to improve product integration and ease-of-use by a field operator. However, although the test specifications require plasma, which necessitates centrifugation of blood, the current device is unlikely to be used widely in a primary care, clinic-based healthcare setting. Although others have been working on a separate POC plasma separation device, which involves its own technical challenges [30], Cepheid has been developing a revised test cartridge to accept a whole blood fingerprick specimen [31], as well as more portable instrumentation [32].

### Clinical impact

As with any new drug, device, or technology, the real-world clinical impact of an intervention on outcome at an individual and population-based level should be evaluated and optimized. Efforts are underway to evaluate the benefits and costs of implementing POC HIV viral load monitoring in HIV clinics. Existing models of chronic HIV care suggest that task shifting to nurses or even lower staff cadres can generate outcomes that are similar (i.e., noninferior) to ART management by physicians or professional nurses [33,34], and endorsed by the WHO [35]. However, a study in Cape Town showed that ‘down referral’ of chronic ART patients to a different site can lead to higher loss to follow-up rates [36]. Therefore, implementing a chronic HIV care model that utilizes an integrated approach of both healthcare workers and integrated POC HIV viral load testing more efficiently, while ensuring quality patient care, may be essential [37].

**FIGURE 1:**
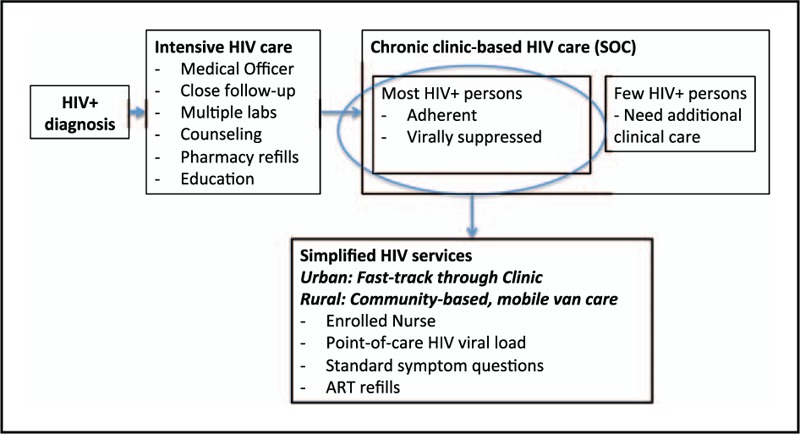
Conceptual model of the Simplified HIV TREtment And Monitoring) study.

A randomized controlled trial, the Simplifying HIV TREatment And Monitoring study, is currently evaluating whether POC HIV viral load monitoring, when combined with nurse-driven task shifting, can improve retention in care and viral load suppression for chronic HIV-infected adults, in a stable ART regimen in a public South African HIV clinic (ref). This study is using a conceptual model that diverts the most stable patients from the acute care pathway and into a more chronic care pathway (Fig. 1). In this model, patients who are ART-adherent and virally suppressed, without substantial ART side effects, will receive simplified HIV care services and rapid HIV viral load testing that could improve engagement in care, increase efficiencies, and reduce costs. Additional studies will be required to determine performance characteristics and optimization in various clinical settings.

The application and adoption of POC technologies to enhancing existing regional and centralized laboratory services is also being evaluated. The Lighthouse Trust (http://mwlighthouse.org/) in Malawi is evaluating whether ‘targeted’ POC viral load testing within a centralized reference laboratory may be beneficial as a confirmatory test. In this case, the POC viral load is used as a second viral load test to confirm treatment failure and help inform the decision to switch to a second-line ART regimen. As POC technology has the rapid turnaround time, an earlier treatment decision may help prevent the spread of HIV drug resistance.

### Integrating laboratory and clinical networks

Country-level decision-makers will determine whether to adopt POC technologies, and if so, which tests to introduce, and where to place new diagnostic tools within the local health system. Those decisions will be based on projected benefits and costs, including price of the instrument, consumables, and service agreements, but will be influenced in part by accurately forecasted test volumes. Regardless, a well functioning laboratory network and integration with clinical services will be a major requirement for any successful POC testing program. Clinical staff will need to be trained on the proper storage, maintenance, and usage of the POC technology, as well as an interpretation of test results and appropriate clinical management. The laboratory network will need to oversee the quality control and assurance of accurate test results and to be aware of any malfunction or broken equipment. This new paradigm for decentralized diagnostic testing will require a more integrated laboratory and clinical network – the essential structure and function of which have been largely undefined.

## REACHING THE LAST MILE: HIV SELF-TESTING

**FIGURE 2:**
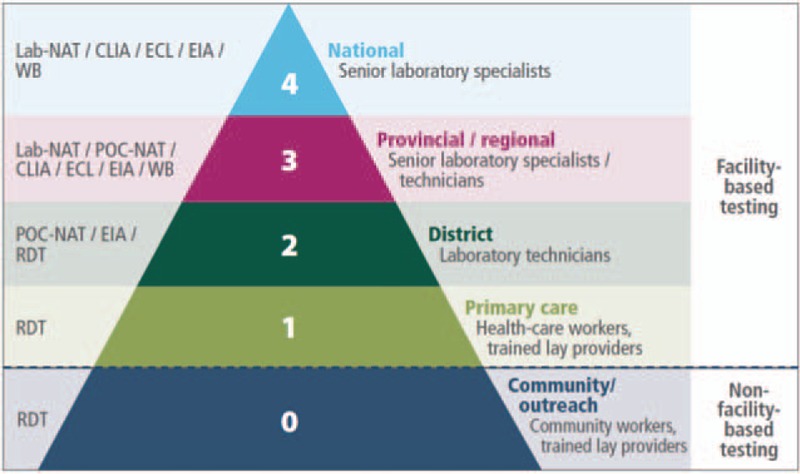
WHO's diagnostic pyramid for HIV testing. ^∗^Adopted from Ref. [8].

The WHO diagnostic pyramid has recently added Level 0 for nonfacility-based testing to expand rapid diagnostic testing in community outreach programs (Fig. 2). HIV self-testing is one innovative strategy that uses POC tests to help achieve the 90-90-90 targets by expanding access to testing services to reach those who may not otherwise test for HIV. As of July 2016, there were four rapid HIV tests in the developed world market with approval for self-testing [38]. This includes three tests for use on fingerstick whole blood and one test for use on oral fluid (Table 1). The DPP HIV 1/2 Assay (Chembio Diagnostic Systems Inc.; Medford, New York, USA) and the OraQuick HIV 1/2 Rapid Antibody Test (OraSure Technologies Inc.; Bethlehem, Pennsylvania, USA) have excellent diagnostic accuracy and have received WHO Prequalification Status for use by a professional clinician. As of October 2016, no tests have received WHO Prequalification for use as a self-test, but this may change in the near future as WHO prepares to review these applications. Several additional HIV rapid diagnostic tests for self-testing are currently in development and may be released in the coming years. One area of concern, however, has been the issue of responsible usage of HIV self-testing within the community.

**Table 1:** Existing rapid HIV diagnostic tests for self-testing available on the market

Assay (manufacturer)	Specimen	Sens./Spec.	Approval status	Price per test
Autotest VIH (AAZ Labs; Rungis-Cedex, France)	Fingerstick whole blood	100%/99.8%	CE marked; WHO PQ submitted	US$25–28
BioSURE HIV Self Test (BioSURE; London, United Kingdom)	Fingerstick whole blood	99.7%/99.9%	CE marked	US$7.5–15
INSTI HIV Self Test (bioLytical Laboratories; Richmond, British Columbia, Canada)	Fingerstick whole blood	100%/99.8%	CE marked	36
OraQuick In-Home HIV Test (OraSure Technologies Inc.; Bethlehem, Pennsylvania, USA)	Oral fluid	100%/99.8%	Pending CE certificate	N/A

Adopted from Ref. [38].

In high-income markets, approximately 1.6 million HIV self-tests have been sold since 2012 [38]. However, the global demand for HIV rapid diagnostic tests for self-testing is uncertain, and one estimate is for 4.8 million tests by the year 2018 [39]. Currently, the price for these tests ($7.50–36/test) appears to be prohibitively high for routine use in low-income and middle-income countries. Although there are many uncertainties about the HIV self-testing market, one commonly cited concern among manufacturers is the poor regulatory and registration processes at the country-level [38]. Reports from Kenya, South Africa, and Namibia suggest that self-tests are being sold from US $1 to 12 per test [40–42]. The development of WHO guidelines and criteria for WHO prequalification would provide some helpful guidance. In addition, we are unaware of any willingness-to-pay surveys from public sector purchasers or program implementation recommendations for low-income and middle-income countries.

Overall, the acceptance among people in low-income and middle-income counties appears to be good. A study of community-based distribution of HIV self-test products in Malawi reported a population-level uptake of 77% over a 2-year period [43]. The self-testing rates were highest among younger age groups, and nearly half of those people testing were first-time testers. In Zimbabwe, a study of community-level distribution found high levels of uptake, including among men, in a rural district [44]. In Kenya, 75–91% of pregnant women and female sex workers, who were offered a self-test kit for their sexual partner, reported secondary distribution of the HIV test kit [45]. Additional studies to evaluate prevention and linkage to care for treatment are currently underway.

## CONCLUSION

The impact of implementing novel or new applications of existing POC diagnostics for HIV will not be fully realized, until the laboratory and clinical networks have been integrated, and the ecosystem is ready. From centralized laboratory systems to POC instrumented technologies to community-based HIV self-tests, diagnostics must be integrated into an effective laboratory system that in turn must be linked to effective healthcare management. The centralized laboratory may serve as the backbone from which an extension of clinic-based POC diagnostic testing and self-testing can occur, which will extend the reach of the centralized laboratory system to meet a large portion of the remaining needs. The operational characteristics and cost-effectiveness of each POC technology will drive placement and adoption, and each country may have unique infrastructure and operational requirements. Ultimately, new POC technologies offer promise, but face significant challenges for improving the system as a whole. The community of global health scientists, developers, clinicians, policy-makers, and economists need to work together in supporting the laboratory and healthcare system holistically to have a transformative effect on improving the clinical services for HIV-infected patients in resource-limited settings.

## Acknowledgements

None.

### Financial support and sponsorship

P.K.D. receives research support from the National Institutes of Health (AI108293, AI124719, and AI127200), the Infectious Disease Society of America, and the Bill and Melinda Gates Foundation.

### Conflicts of interest

There are no conflicts of interest.

## REFERENCES AND RECOMMENDED READING

Papers of particular interest, published within the annual period of review, have been highlighted as:

▪ of special interest
▪▪ of outstanding interest
